# Intravenous Administration of Cilostazol Nanoparticles Ameliorates Acute Ischemic Stroke in a Cerebral Ischemia/Reperfusion-Induced Injury Model

**DOI:** 10.3390/ijms161226166

**Published:** 2015-12-09

**Authors:** Noriaki Nagai, Chiaki Yoshioka, Yoshimasa Ito, Yoshinori Funakami, Hiroyuki Nishikawa, Atsufumi Kawabata

**Affiliations:** Faculty of Pharmacy, Kinki University, 3-4-1 Kowakae, Higashi-Osaka, Osaka 577-8502, Japan; nagai_n@phar.kindai.ac.jp (N.N.); 1444420001d@kindai.ac.jp (C.Y.); funakami@phar.kindai.ac.jp (Y.F.); h-nishikawa@fuso-pharm.co.jp (H.N.); kawabata@phar.kindai.ac.jp (A.K.)

**Keywords:** cilostazol, ischemic stroke, injection formulation, nanoparticles, pharmacokinetics

## Abstract

It was reported that cilostazol (CLZ) suppressed disruption of the microvasculature in ischemic areas. In this study, we have designed novel injection formulations containing CLZ nanoparticles using 0.5% methylcellulose, 0.2% docusate sodium salt, and mill methods (CLZ_nano_ dispersion; particle size 81 ± 59 nm, mean ± S.D.), and investigated their toxicity and usefulness in a cerebral ischemia/reperfusion-induced injury model (MCAO/reperfusion mice). The pharmacokinetics of injections of CLZ_nano_ dispersions is similar to that of CLZ solutions prepared with 2-hydroxypropyl-β-cyclodextrin, and no changes in the rate of hemolysis of rabbit red blood cells, a model of cell injury, were observed with CLZ_nano_ dispersions. In addition, the intravenous injection of 0.6 mg/kg CLZ_nano_ dispersions does not affect the blood pressure and blood flow, and the 0.6 mg/kg CLZ_nano_ dispersions ameliorate neurological deficits and ischemic stroke in MCAO/reperfusion mice. It is possible that the CLZ_nano_ dispersions will provide effective therapy for ischemic stroke patients, and that injection preparations of lipophilic drugs containing drug nanoparticles expand their therapeutic usage.

## 1. Introduction

Strokes lead to a high level of disability and are a leading cause of death worldwide. The number of patients suffering ischemic strokes resulting from nonvalvular atrial fibrillation, the major cause of ischemic stroke, is predicted to double by 2030 [1]. Cerebral ischemia and reperfusion injury lead to damage of brain tissues, inflammation as a result of the disruption of the blood–brain barrier (BBB), oxidative damage [2], and apoptosis [3]. Overall, excitotoxicity, inflammation and oxidative stress play crucial roles in the pathophysiology of ischemic stroke [4,5]. In acute ischemic stroke, the re-establishment of cerebral blood flow was caused by the treatment of tissue plasminogen activator (t-PA) within 4.5 h after the symptom onset, and the intravenous administration with recombinant t-PA (rt-PA) within a few hours after the onset of ischemic attack has also been demonstrated by the National Institute of Neurological Disorders and the Stroke rt-PA Stroke Study Group [6]. Although, the t-PA is very useful in therapy for ischemic stroke patients, it is known that the t-PA causes the tPA-induced cell damage in human brain microvascular endothelial cells [7]. Therefore, further study of the development of new therapies for ischemic stroke patients is important.

Cilostazol (CLZ, 6-[4-(1-cyclohexyl-1H-tetrazol-5-yl)butoxy]-3,4-dihydrocarbostyril) is an inhibitor of phosphodiesterase-III, which increases intracellular cyclic adenosine monophosphate (AMP) levels by restraining platelet aggregation [8]. It is widely used for the secondary prevention of cerebral infarction and treatment for intermittent claudication in peripheral artery disease. In addition to its anti-platelet effect, the CLZ shows the inhibition of apoptosis and endothelial protection in endothelial cells [9] prevented the phenotypic modulation in vascular smooth muscle cells [10], and sustains cerebral blood flow (CBF) by endothelium-independent vasodilation [11]. Further, it has been reported that CLZ protects the tPA-induced cell damage in human brain microvascular endothelial cells [7], and the CLZ can attenuate ischemic brain injury by maintaining endothelial function in the cerebral cortex [12]. Kasahara *et al.* [13] also reported that treatment with CLZ suppresses the disruption of the microvasculature in ischemic areas, and Hara *et al.* showed that CLZ reduces hemorrhagic transformations after cerebral ischemia by preventing BBB disruption in mice [14]. These findings suggest that the administration of CLZ may effectively attenuate the brain damage by the treatment immediately after the symptom onset of ischemic stroke. Therefore, the development of drug delivery systems (DDS) for the intravenous administration of CLZ is desirable.

It is expected that drug systems using nanoparticles will improve the problem of poor water solubility in injectable preparations of lipophilic drugs. Nano-sized particles such as liposomes [15], polymer micelles [16], emulsions [17] and poly (lactic-coglycolic acid) (PLGA) nanospheres [18,19] have been widely used in DDS, and the DDS enhanced drug absorption and reduced side-effects. Furthermore, PLGA nanospheres can be applied to intravenous drug delivery systems [20]. On the other hand, we have also developed methods to prepare drug nanoparticles by a bead mill method [21,22]. Here, we have designed novel injection formulations containing CLZ nanoparticles, and investigated their safety (toxicity) by measuring hemolysis, blood pressure and blood flow. In addition, we demonstrate the protective effect of the intravenous injection of dispersions containing CLZ nanoparticles in ischemic stroke using a cerebral ischemia/reperfusion-induced injury model (MCAO/reperfusion mice).

## 2. Results

### 2.1. Evaluation of Particles Size and Stability of Dispersions Containing CLZ Nanoparticles

**Figure 1:**
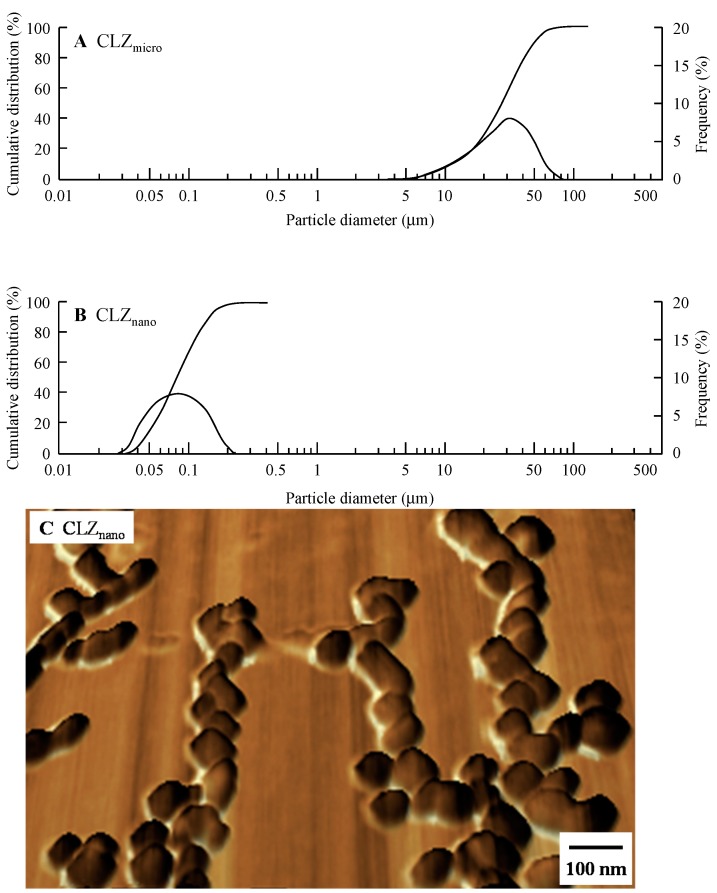
Cumulative size distribution, frequency and images of dispersions containing CLZ nanoparticles. (**A**) Cumulative size distribution and frequency of CLZ_micro_ dispersion; (**B**) Cumulative size distribution and frequency of CLZ_nano_ dispersion; (**C**) Image of the CLZ_nano_ dispersion under the SPM-9700. The compositions of the preparations containing CLZ are shown in Table 1. Particle size and the image of the CLZ dispersions were determined using a nanoparticle size analyzer SALD-7100 and an SPM-9700, respectively.

Figure 1 shows the particle size distribution of dispersions containing CLZ as described in Table 1. The mean particle size using untreated CLZ (CLZ_micro_) was 24.7 ± 14.3 μm (mean ± S.D.), and reached a gel-like state when subjected to the mill method. On the other hand, CLZ particles (Milled-CLZ_MC_) obtained by adding methylcellulose (MC) and using the mill method had a mean particle size of 83 ± 62 nm (means ± S.D.). The mean particle size for CLZ subjected to the mill method in the presence of both MC and docusate sodium salt (DS) was 81 ± 59 nm (CLZ_nano_, mean ± S.D.); the addition of DS alone did not affect the mean particle size.

**Table 1:** Formulations of particle dispersions containing CLZ.

Formulation	Content (*w*/*v* %)			Treatment
	CLZ Microparticles	MC	DS	
CLZ_micro_	0.5	0.5	0.2	—
Milled-CLZ_MC_	0.5	0.5	—	Ball mill + Bead mill
Milled-CLZ_DS_	0.5	—	0.2	Ball mill + Bead mill
CLZ_nano_	0.5	0.5	0.2	Ball mill + Bead mill

The particle sizes of CLZ dispersions as shown in Table 1 were measured by a SALD-7100. The data are presented as means ± S.D.

**Figure 2:**
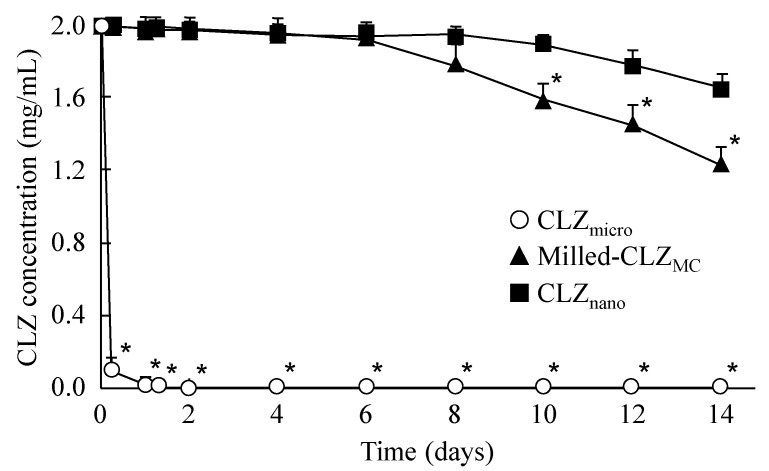
Stability of 0.5% CLZ dispersions as shown in Table 1. The experiment was done in the dark place (20 °C, 14 days). Thedata are presented as means ± S.E. of five independent samples. * *p* < 0.05, *vs.* CLZ_nano_.

Figure 2 shows the stability of dispersions containing 0.5% CLZ as described in Table 1. The CLZ_micro_ preparation precipitated 4 h after preparation; the stability was increased by the combination of added MC and the mill method (Milled-CLZ_MC_). Furthermore, the addition of DS further enhanced the stability of the CLZ dispersion (CLZ_nano_), and no precipitation was observed 14 days after preparation. Table 2 shows the changes in CLZ particle size 14 days after preparation. Although the particle size of Milled-CLZ_MC_ increased to 0.52 ± 0.23 μm from 0.083 ± 0.062 μm, there was no change in the particle size of CLZ_nano_ between 0 and 14 days after preparation.

**Table 2:** Changes in CLZ particle size in CLZ dispersions 14 days after treatment with ball and bead mill.

Formulation	Particle Size (μm)	
	Immediately	14 Days after Preparation
CLZ_micro_	24.7 ± 14.3	25.1 ± 14.9
Milled-CLZ_MC_	0.083 ± 0.062	0.52 ± 0.23
CLZ_nano_	0.081 ± 0.059	0.086 ± 0.065

### 2.2. Evaluation of the Safety of Intravenous Injections of Dispersions Containing CLZ Nanoparticles

**Figure 3:**
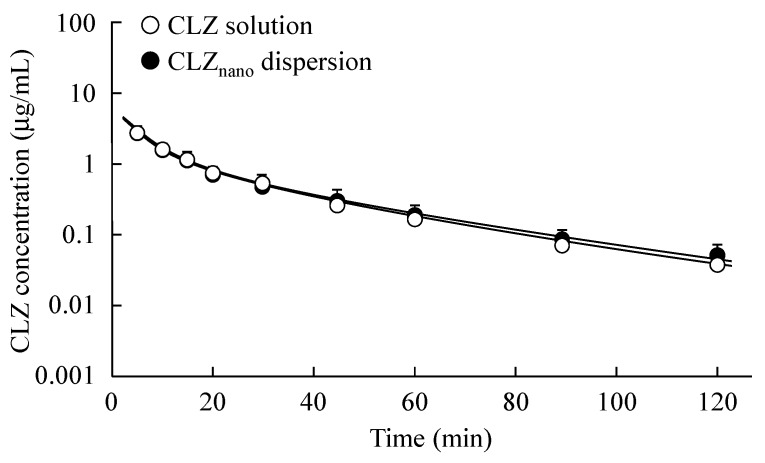
Changes in plasma CLZ concentration after intravenous injection of CLZ solution or CLZ_nano_ dispersion. 0.3 mL of CLZ solution (open circles) or CLZ_nano_ dispersion (closed circles) was injected into the femoral vein (0.1 mg/kg), and venous blood (200 μL) was collected. Solid lines present fitting curves for plasma CLZ fates after intravenous administration of CLZ solution or CLZ_nano_ dispersion. The data are presented as means ± S.E. of seven independent rats.

Figure 3 shows the changes in plasma CLZ concentration of rats treated with CLZ solution or dispersions containing CLZ nanoparticles (0.1 mg/kg), and Table 3 shows the pharmacokinetic parameters of the intravenous injection of the CLZ solution or dispersions containing CLZ nanoparticles. There were no significant differences in the elimination rate constants (α and β) of the CLZ solution and dispersions containing CLZ nanoparticles. In addition, the area under the CLZ concentration-time curve (*AUC*_CLZ_) and mean residence time (*MRT*_CLZ_) of dispersions containing CLZ nanoparticles was also similar to those of the CLZ solution. In this study, we investigated the effect of dispersions containing 0.5% CLZ nanoparticles on the rate of hemolysis of rabbit red blood cells (RBC), a model of cell injury. Hemolysis was observed for preparations containing 2-hydroxypropyl-β-cyclodextrin at concentrations over 0.6% (0.6%: 6.1% ± 0.9%, 1.0%: 57.2% ± 1.4%, *n* = 5). On the other hand, the RBC membranes were not broken by treatment with dispersions containing 0.5% CLZ nanoparticles for 30 min [hemolysis rate of 1.9% ± 0.6% (*n* = 5)].

Parameters were calculated according to Equations (4)–(6) (see Experimental Section). The data are presented as means ± S.E. of seven independent rats.

**Figure 4:**
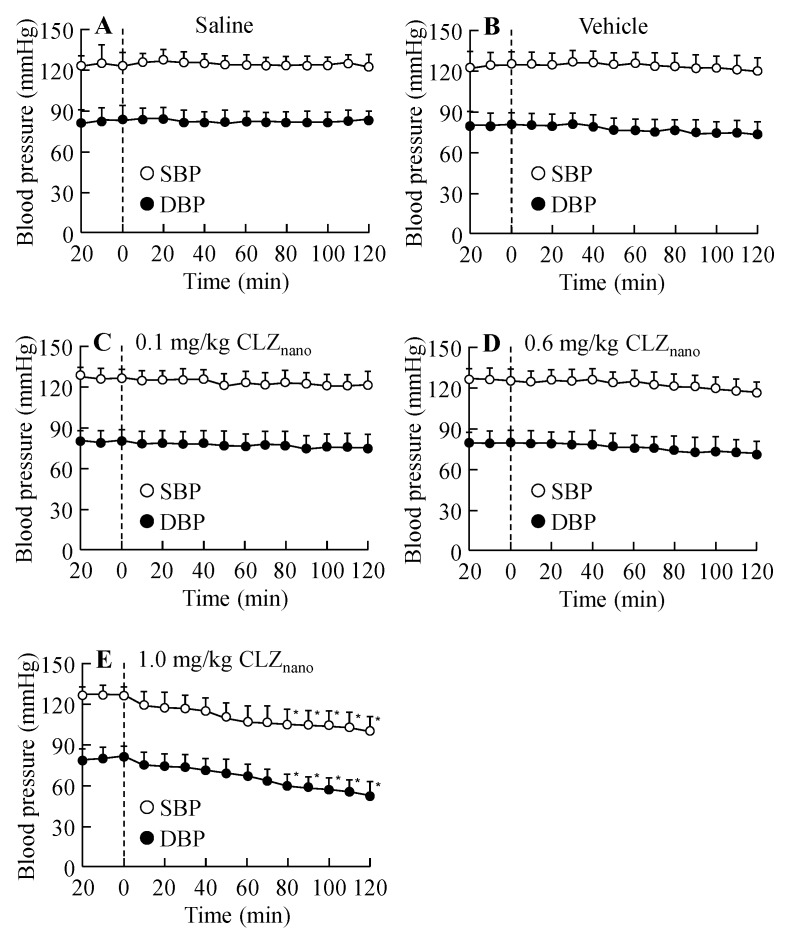
Changes in blood pressure following the intravenous injection of CLZ_nano_ dispersion. Saline (**A**); vehicle (**B**); 0.1 mg/kg CLZ_nano_ (**C**); 0.6 mg/kg CLZ_nano_ (**D**) or 1.0 mg/kg CLZ_nano_ (**E**) dispersion was injected into the tail vein. The rats were anesthetized with Urethane (1.2 g/kg, i.p.), and the systolic blood pressure (SBP, open circles) and diastolic blood pressure (DBP, closed circles) were measured using a transducer (DX312) for 140 min (11:00–13:20). CLZ was injected 20 min after the first measurement (0 min). The data are presented as means ± S.E. of seven independent rats. * *p* < 0.05, *vs.* blood pressure at 0 min for each category.

**Figure 5:**
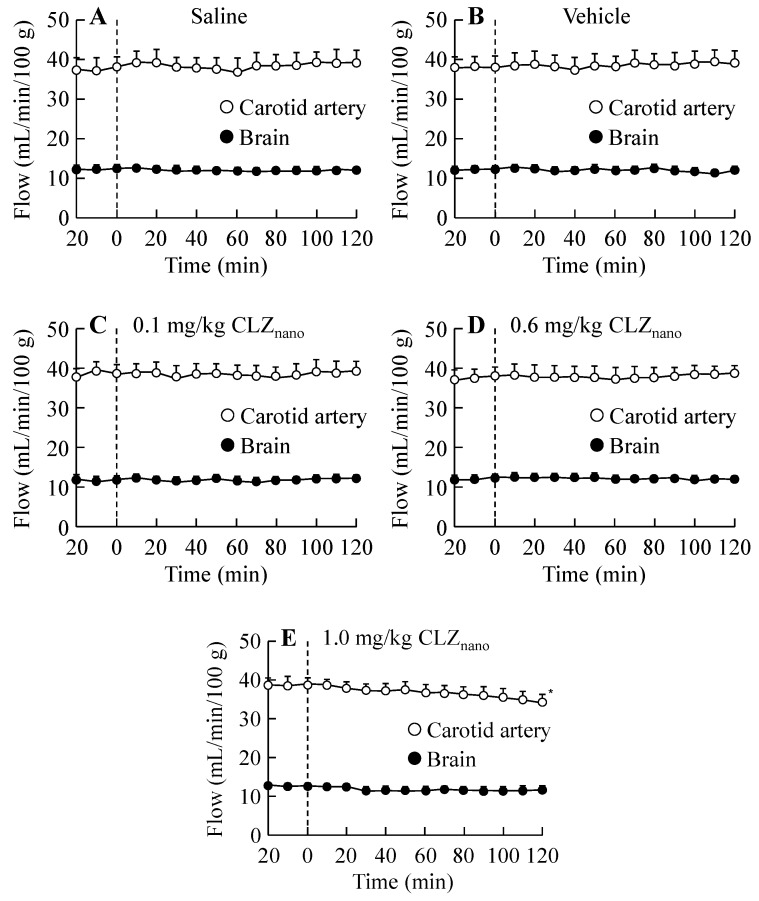
Changes in blood flow following the intravenous injection of CLZ_nano_ dispersion. Saline (**A**); vehicle (**B**); 0.1 mg/kg CLZ_nano_ (**C**); 0.6 mg/kg CLZ_nano_ (**D**) or 1.0 mg/kg CLZ_nano_ (**E**) dispersion was injected into the tail vein. The rats were anesthetized with Urethane (1.2 g/kg, i.p.), and the blood flow in the carotid artery (open circles) and brain (closed circles) were measured using a non-contact type laser doppler flowmeter (OMEGA Flow FLO-N1). CLZ was injected 20 min after the first measurement (0 min). The data are presented as means ± S.E. of seven independent rats. * *p* < 0.05, *vs.* blood flow at 0 min for each category.

Figure 4 and Figure 5 show the effects on blood pressure (Figure 4) and blood flow in the carotid artery (Figure 5) in rats treated with dispersions containing CLZ nanoparticles. No changes were observed in the blood pressure or blood flow in the carotid artery of rats receiving an intravenous injection of 0.1 mg/kg or 0.6 mg/kg CLZ nanoparticle dispersions; however, the intravenous injection of 1.0 mg/kg CLZ dispersions containing nanoparticles decreased both blood pressure and blood flow. On the other hand, the blood flow in the brain was not changed by the intravenous injection of 0.1–1.0 mg/kg CLZ dispersions containing nanoparticles (Figure 5), and the intravenous injection of 0.1–1.0 mg/kg CLZ dispersions containing nanoparticles for one week (once a day) had no effect on body weight or mortality.

**Table 3:** Pharmacokinetic parameters of plasma CLZ concentration after intravenous injection of CLZ solution and CLZ_nano_ dispersion.

	*A* (μg/mL)	*A* (/min)	*B* (μg/mL)	β (×10^−2^, /min)	*AUC*_CLZ_ (μg·min/mL)	*MRT*_CLZ_ (min)
CLZ solution	3.87 ± 0.27	0.16 ± 0.01	1.27 ± 0.12	3.41 ± 0.52	60.0 ± 6.9	21.6 ± 1.7
CLZ_nano_ dispersion	3.85 ± 0.41	0.17 ± 0.01	1.31 ± 0.10	3.08 ± 0.24	57.1 ± 8.1	24.1 ± 2.7

### 2.3. Protective Effect of the Intravenous Injection of Dispersions Containing CLZ Nanoparticles on Ischemic Stroke in MCAO/Reperfusion Mice

**Figure 6:**
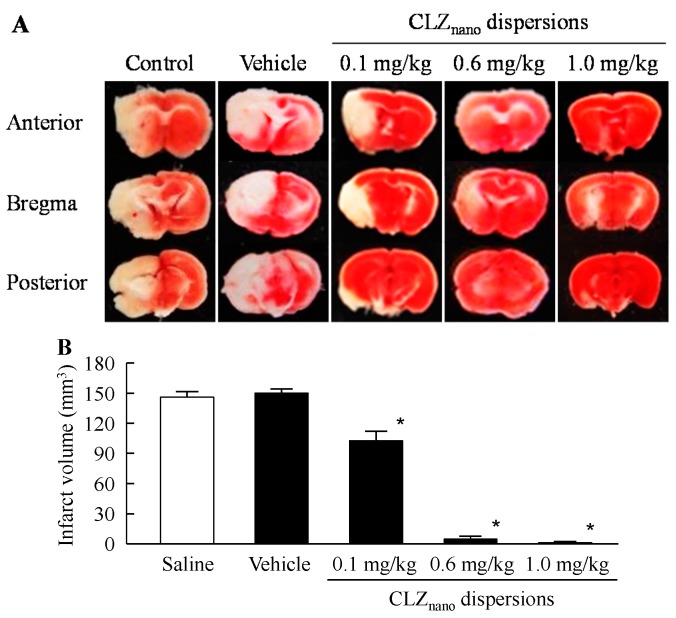
Protective effect of dispersions containing CLZ nanoparticles on ischemic stroke in MCAO/reperfusion mice. (**A**) Image of the brain (the bregma and 1 mm anterior and 1 mm posterior to the bregma) of MCAO/reperfusion mice treated with CLZ_nano_ dispersion; (**B**) Infarct volume of MCAO/reperfusion mice treated with CLZ_nano_ dispersion. MCAO was induced by inserting a silicone-coated 8-0 nylon monofilament into the left middle cerebral artery. Two hours after this procedure, the middle cerebral artery blood flow was restored by withdrawing the nylon monofilament. At 3 h after reperfusion, saline, vehicle, 0.1 mg/kg CLZ_nano_, 0.6 mg/kg CLZ_nano_ or 1.0 mg/kg CLZ_nano_ dispersion was injected into the tail vein; three days after injection, the brains were removed. The data are presented as means ± S.E. of seven independent mice. * *p* < 0.05, *vs.* Saline.

Figure 6 shows the changes in ischemic stroke in MCAO/reperfusion mice treated with dispersions containing CLZ nanoparticles. Ischemic stroke was not observed in the sham-control mice (data not shown). MCAO/reperfusion induced ischemic stroke, and the infarct area and volume in MCAO/reperfusion mice treated vehicle was 26.7 ± 1.2 mm^2^ and 149.9 ± 6.2 mm^3^, respectively (means ± S.E., *n* = 7). The infarct volume in the MCAO/reperfusion mice treated with saline was higher than that in previous reports [23]. The infarct volume is changed by the measuring point used for the calculation, and the number of points used for measurement in this study (three point, Area_a-c_) was few in comparison with that in the previous report of Carmichael [23]. This methodological difference (the difference in number of measuring points) may be related to the high infarct volumes in this study. The injection of dispersions containing CLZ nanoparticles attenuated ischemic stroke, and the protective effect increased with CLZ concentration. In addition, the neurological deficits in MCAO/reperfusion mice were also ameliorated by the injection of dispersions containing CLZ nanoparticles (saline 2.7 ± 0.2, 0.6 mg/kg CLZ_nano_ 1.1 ± 0.3, means ± S.E., *n* = 7). On the other hand, the infarct volume of MCAO/reperfusion mice treated with CLZ_nano_ dispersion (104.3 ± 9.6 mm^3^, means ± S.E., *n* = 7) was similar to that in 0.1 mg/kg CLZ solution (113.1 ± 7.6 mm^3^, means ± S.E., *n* = 5). In addition, we evaluated the protective effect of a commercially available CLZ OD (oral dispersing) tablet on ischemic stroke in MCAO/reperfusion mice. Although, the oral administration of commercially available CLZ OD tablet attenuated ischemic stroke, the infarct volume of MCAO/reperfusion mice treated with the commercially available CLZ OD tablet (3 mg/kg 81.9 ± 8.7 mm^3^, 30 mg/kg 18.3 ± 2.1 mm^3^, means ± S.E., *n* = 4–8) was higher than that treated with 0.6–1.0 mg/kg CLZ dispersions containing nanoparticles.

**Figure 7:**
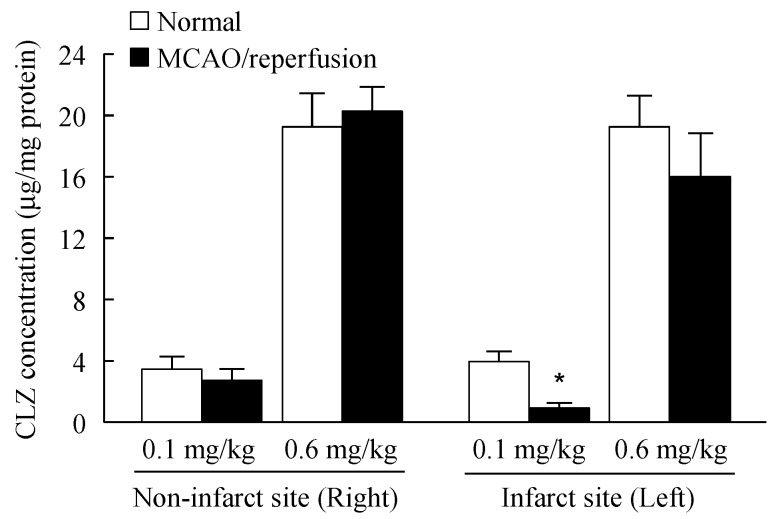
CLZ concentration in the brains of normal and MCAO/reperfusion mice treated with dispersions containing CLZ nanoparticles. MCAO was induced by inserting a silicone-coated 8-0 nylon monofilament into the left middle cerebral artery. Two hours after this procedure, the middle cerebral artery blood flow was restored by withdrawing the nylon monofilament. At 3 h after reperfusion, 0.1 or 0.6 mg/kg CLZ_nano_ dispersion was injected into the tail vein. Three days after the injection, the brains were removed, and cut 1 mm anterior and 1 mm posterior to the center of the bregma. The sliced brain tissue was divided at the center, and CLZ concentrations in the right (non-infarct site) and left (infarct site) parts were measured. The data are presented as means ± S.E. of seven independent rats. * *p* < 0.05, *vs.* normal mice for each category.

Figure 7 shows the CLZ concentration in the brain of MCAO/reperfusion mice treated with dispersions containing CLZ nanoparticles. CLZ was detected in the brain of MCAO/reperfusion mice, and the concentrations were similar between normal and MCAO/reperfusion mice in the right side (non-infarct site). In the left side (infarct site), the CLZ concentration in MCAO/reperfusion mice treated with 0.1 mg/kg dispersions containing CLZ nanoparticles was significantly lower than in normal mice. On the other hand, there were no significant differences in the CLZ concentration between the normal and MCAO/reperfusion mice treated with 0.6 mg/kg dispersions containing CLZ nanoparticles on either side. We also found the blood flow in the brains of MCAO/reperfusion mice was lower than in normal mice (normal mice 10.8 ± 0.8, MCAO/reperfusion mice 7.2 ± 0.8 mL/min/100 g, means ± S.E., *n* = 7), while the blood flow in the brains of MCAO/reperfusion mice treated with 0.6 mg/kg CLZ dispersions containing nanoparticles (10.1 ± 1.0 mL/min/100 g, means ± S.E., *n* = 7) was higher than that in the untreated MCAO/reperfusion mice.

## 3. Discussion

In this study, we designed novel injection formulations containing CLZ nanoparticles, and investigated their safety, toxicity and usefulness using a cerebral ischemia/reperfusion-induced injury model (MCAO/reperfusion mice).

We attempted to mill CLZ microparticles further by the ball and bead mill methods, but the CLZ reached a meringue state (Milled-CLZ); therefore, we searched for new innovations to prepare CLZ nanoparticles. MC is a derivative of cellulose, and is highly biocompatible [24,25] and used in the preparation of drug formulations. We previously reported that the addition of MC enhances the crushing efficiency in the bead mill method [21,22]. In this study, the particle size of CLZ was decreased using a combination of MC addition and the mill method, with the CLZ particles obtained having a mean particle size of 83 ± 62 nm (Milled-CLZ_MC_, mean ± S.D.).

Next, the stability of CLZ dispersions was measured. The precipitation in CLZ_micro_ preparation was observed 4 h after preparation. The stability was improved by the combination of MC addition and the mill method; however, precipitation was still observed 10 days after preparation (Figure 2 and Table 2). DS are surface-active agents, and we previously reported that the addition of surface-active agents prevents precipitation [21,22]. Therefore, we prepared a CLZ dispersion with 0.5% MC and 0.2% DS by the mill method. The CLZ dispersion was enhanced by the addition of DS (CLZ_nano_, Table 2), and the addition of DS was also increased the CLZ recovery percentage in the mill method (Milled-CLZ_MC_ 84.6% ± 4.7%, CLZ_nano_ 94.3% ± 4.2%, means ± S.E., *n* = 5). In addition, we investigated RBC hemolysis because formulations that cause hemolysis are not suitable for use in injection preparations. The solution containing CLZ prepared with dimethyl sulfoxide caused RBC hemolysis, and the low concentration of dimethyl sulfoxide without hemolysis was not able to reach a CLZ concentration as high as the injection preparations for therapy of ischemic stroke. Hemolysis was also observed for preparations containing 2-hydroxypropyl-β-cyclodextrin at concentrations over 0.6%, and solutions containing 0.008% CLZ can be prepared using 0.6% 2-hydroxypropyl-β-cyclodextrin, however, this CLZ concentration is not therapeutically effective for ischemic stroke. These results show that the CLZ solutions containing dimethyl sulfoxide and cyclodextrin are not suitable for injection preparations. On the other hand, no RBC hemolysis was observed for 0.5% dispersions containing CLZ nanoparticles. The CLZ_nano_ dispersion consists of safe additives (do not use the DMSO and cyclodextrin), and the concentration of additives is lower. These safe formulations may be related to the non-hemolysis of CLZ_nano_ dispersion. Therefore, care should be taken concerning the methods and additives used to prepare nanoparticles in order to find suitable formulations in terms of safety and stability.

In this study, we investigated the toxicity of dispersions containing CLZ nanoparticles by measuring the plasma CLZ concentration, blood pressure and blood flow in rats to confirm the safety of the injection preparations. The particle size influences its fate in the body, and it has been reported that the particles of ≥2 μm were trapped inside liver cells, and the particles of ≥300–400 nm were captured by macrophages and excreted. In addition, the particles of ≥200 nm were filtered out in the spleen, and the particles of ≥100 nm were passed through the blood vessels via the endothelial lining. Thus, the particle size affects functionality, residence in circulation, adherence, degradation, and clearance [26,27,28,29]. The pharmacokinetic parameters for the intravenous injection of dispersions containing CLZ nanoparticles are similar to those of the CLZ solution (Figure 3 and Table 3). Although intravenous injections of CLZ dispersions containing microparticles for one week (once a day) cause the decrease in body weight or death, the intravenous injections of CLZ dispersions containing nanoparticles for one week (once a day) had no effects on body weight or survival. It is known that solubility is enhanced by the preparation of nanoparticles because nanoparticles have a high surface area. In addition, the CLZ interferes with platelet aggregation via an inhibitor of phosphodiesterase-III [8]. Taken together, it is suggested that CLZ nanoparticles in CLZ_nano_ dispersions may dissolve immediately after injection. Moreover, the intravenous injection of CLZ dispersions containing nanoparticles appears not to cause thrombus formation. On the other hand, although the intravenous injection of 1.0 mg/kg CLZ dispersions containing nanoparticles decreases blood pressure and blood flow, blood pressure and blood flow in rats treated with intravenous injections of 0.1 or 0.6 mg/kg CLZ dispersions containing nanoparticles do not change. These results show that while the injection of 1.0 mg/kg CLZ dispersions containing nanoparticles may cause toxicity, the injection of 0.6 mg/kg CLZ dispersions containing nanoparticles is safe.

Many studies have indicated that CLZ treatment suppresses the disruption of the microvasculature in ischemic areas [13], and that CLZ reduces hemorrhagic transformations after cerebral ischemia by preventing BBB disruption in mice [14]. We also investigated the therapeutic effects of CLZ in ischemic stroke in MCAO/reperfusion mice treated with novel CLZ_nano_ dispersions. The intravenous administration of CLZ_nano_ dispersions ameliorates the neurological deficits following ischemic stroke in MCAO/reperfusion mice, and the effects increase with the CLZ concentration. Moreover, CLZ_nano_ dispersions attenuate hypovolemia caused by MCAO/reperfusion in the brain. In addition, the protective effect of CLZ dispersions containing nanoparticles was significantly higher than that of the commercially available CLZ OD tablet in the ischemic stroke of MCAO/reperfusion mice. These results show that the intravenous administration of CLZ_nano_ dispersions may provide therapy for ischemic stroke.

It is important to clarify the mechanisms by which CLZ enables a decrease in the neurological deficits caused by ischemic stroke. It is known that CLZ inhibits platelet aggregation caused by a variety of stimuli, including adenosine diphosphate (ADP), arachidonic acid, epinephrine, collagen, thrombin, and high shear stress [30,31,32]. Because of its unique mechanisms of action, a wide variety of pharmacological actions has been reported, including antithrombosis in feline cerebral ischemia [12], increased cerebral blood flow [33], and vasodilation via an increased cyclic AMP level [12]. Moreover, it has been reported that in rat brains subjected to MCAO followed by 24 h reperfusion, the oral administration of aspirin (300 mg/kg) has little effect on the cerebral infarct size, but that the size is significantly reduced by CLZ (30 mg/kg) [34]. In addition, it was reported that the neuroprotective potential of CLZ is based on its anti-inflammatory and anti-apoptotic effects (mediated by the scavenging of hydroxyl radicals), the decreased formation of tumor necrosis factor-α, the inhibition of poly (ADP-ribose) polymerase activity, and the induction of metallothionein-1 and -2 in brain [35,36,37]. Therefore, the protective effects of CLZ_nano_ dispersions may also be related to these unique mechanisms. On the other hand, the infarct volume indicates that all untreated animals lost more than half of the entire hemisphere while the treated mice had very small infarcts (the damage in infarct core was also improved). The damage of brain in MCAO/reperfusion mice was enhanced by the reperfusion, and the mice in this study were treated for three days after reperfusion. Therefore, the CLZ prevents the exacerbation of damage by reperfusion, and the permanent damage in the infarct core is lower in this model. In addition, the permanent damage in the infarct core may be not detected by the method using *2*,*3*,*5*-triphenyl tetrazolium chloride (condition: 1.5%, 15 min), since the damage is lower in this model. On the other hand, the location of measurement and distance between slices (2 mm) of brain tissue may also be related to the apparent disappearance of damage in the infarct core.

Further studies are needed to elucidate the usefulness and toxicity of the intravenous administration of dispersions containing CLZ nanoparticles as therapy for ischemic stroke. In addition, it is important to clarify the relationships between the timing of administration and curative effect on ischemic stroke. Therefore, we are now investigating the changes in ischemic stroke when the CLZ_nano_ dispersions are administered 6–24 h after MCAO/reperfusion. Hara *et al.* reported that CLZ protects against subacute tPA-induced cerebral injury in mice that have undergone hemorrhagic transformation, and inhibits tPA-induced cell damage in human brain microvascular endothelial cells [7]. Therefore, we will also demonstrate the usefulness of CLZ_nano_ dispersions in tPA-administered mice.

## 4. Materials and Methods

### 4.1. Animals and Materials

Male Wistar rats aged 7 weeks, ICR mice aged 5 weeks, and rabbits, 2.5–3.0 kg, were used, and housed under conditions: 12 h/day fluorescent light (07:00–19:00), 25 °C room temperature. All animal studies were performed following the Kinki University Faculty of Pharmacy Committee Guidelines for the Care and Use of Laboratory Animals. Low-substituted methylcellulose (MC, METOLOSE SM-4, average viscosity, 4 Pa·s at 20 °C) was purchased from Nihon Shokuhin Kako Co., Ltd. (Tokyo, Japan). Conventional CLZ (solid, CLZ microparticles, 22.8 ± 14.1 μm, means ± S.D.) was obtained from Otsuka Pharmaceutical Co., Ltd. (Tokushima, Japan). Docusate sodium salt (DS) was purchased from Sigma-Aldrich Japan (Tokyo, Japan). All other chemicals used were of the highest purity commercially available. In this study, experiments were performed blind for treatment group.

### 4.2. Preparation of Dispersions Containing CLZ Nanoparticles

CLZ nanoparticles were prepared using zirconia balls, Pulverisette 7 (a planetary ball *mill*, *Fritsch* Japan Co., Ltd., Tokyo, Japan) and Bead Smash 12 (a bead mill, Wakenyaku Co., Ltd, Kyoto, Japan). Zirconia balls (diameter: 10 mm) were added to a zirconia cup (diameter: 45 mm) containing CLZ microparticles, and the mixture was crushed with the Pulverisette 7 for 24 h (400 rpm, room temperature). The mixtures were then dispersed in sterilized isotonic saline containing MC or DS, and crushed again with the Bead Smash 12 (5500 rpm, 30 s × 15 times, 4 °C) in the presence of zirconia beads (diameter: 0.1 mm). These preparations were done under aseptic conditions, and the formulations of the CLZ dispersions are presented in Table 1. The pH of dispersions containing CLZ micro- or nanoparticles was 6.7. The particle sizes and images were obtained using a nanoparticle size analyzer SALD-7100 (Shimadzu Corp., Kyoto, Japan; refractive index 1.60-0.10i) and scanning probe microscope SPM-9700 (Shimadzu Corp., Kyoto, Japan), respectively. The image of dispersions containing CLZ nanoparticles (CLZ_nano_) as described in Table 1 was created by combining a phase and height image using image analysis software connected to the SPM-9700. CLZ solubility in saline containing 0.5% MC and 0.2% DS was not detected by the high performance liquid chromatography (HPLC) method (solubility was detected using a 0.22 μm membrane filter). In this study, we used the solution containing CLZ (CLZ solution) as a control. A 0.008% CLZ solution was prepared in 0.6% 2-hydroxypropyl-β-cyclodextrin as described in our previous our report [38]. In this preparation, the solvent containing the additives was filtered through a Minisart CE (pore size 0.20 μm, Costar, Cambridge, MA, USA).

### 4.3. Stability of Dispersions Containing CLZ

CLZ (3 mL) dispersions as shown in Table 1 were incubated in test tubes (5 mL) in dark condition (20 °C for 14 days), after which sample solutions (50 μL) were taken from 5 mm under the surface (the liquid surface height is 4 cm). The CLZ concentrations in the samples were determined by the following HPLC method. Fifty microliters of sample was added to 50 μL methanol containing 0.25 μg indomethacin (internal standard), and the mixture was filtered through a Chromatodisk 4A (pore size 0.45 μm, Kurabo Industries Ltd., Osaka, Japan). The solution (10 μL) was used, and measured by using a Shimadzu LC-20AT system (Shimadzu Corp., Kyoto, Japan) equipped with an Inertsil^®^ ODS-3 (3 μm, column size: 2.1 mm × 50 mm) column (GL Science Co., Inc., Tokyo, Japan). The mobile phase consisted of acetonitrile/methanol/water (35/15/50, *v*/*v*/*v*) at a flow rate of 0.25 mL/min; the column temperature was 35 °C, and the wavelength for detection was 254 nm.

### 4.4. Hemolysis of Rabbit Red Blood Cells (RBC) by Treatment with Dispersions Containing CLZ Nanoparticles

Blood was collected from the marginal ear vein of adult Japanese albino rabbits weighing 2.5–3.0 kg, and 1 mL blood was mixed with 100 mL heparin (10 mg/mL). The mixture was centrifuged at 3000 rpm for 5 min at 37 °C, and the pellets were washed with phosphate buffered saline (pH 7.4). The resulting pellets were used as red blood cells (RBC) in this study. Forty μL RBC was incubated with dispersions containing CLZ nanoparticles for 30 min. After incubation, the RBC solutions were centrifuged at 2300 rpm for 5 min at 37 °C, and absorbance (Abs) at 576 nm was measured. The rate of hemolysis was calculated according to Equation (1):

(1) Rate of hemolysis (%) = Abs_treatment_/Abs_non-treatment_ × 100

### 4.5. Induction of Focal Cerebral Ischemia/Reperfusion

Focal cerebral ischemia/reperfusion was caused by middle cerebral artery occlusion (MCAO) completed following the methods of Hara *et al.* [39]. The mice were anesthetized by isoflurane (2.5%) using BS-400T (Brain Science Idea Co., Ltd., Osaka, Japan), and the body temperature was regulated at 37 °C. MCAO was induced by insertion of a silicone-coated 8-0 nylon monofilament (Natsume Seisakusyo Co., Ltd., Tokyo, Japan) into the left middle cerebral artery. Two hour after this procedure, the mice were briefly reanesthetized with isoflurane, and the middle cerebral artery blood flow was restored by withdrawing the nylon monofilament. Sham-control mice underwent the same surgical procedure but without middle cerebral artery obstruction. At 3 h after reperfusion, a dose of 0.1, 0.6 or 1.0 mg/kg of CLZ_nano_ was injected into the tail vein using a 1 mL syringe and a 27-gauge needle. The injection volume was set at 5 mL/kg body weight. The vehicle-treated mice were injected with the same volume of saline containing 0.5% MC and 0.2% DS. Three days after reperfusion, the brain was removed and three slices were cut out for analysis: from the bregma (2 mm, Area_A_), 1 mm anterior to the bregma (2 mm, Area_B_), and 1 mm posterior to the bregma (2 mm, Area_C_), using Brain Matrices (Brain ScienceIdea Co., Ltd., Osaka, Japan). The slices of brain tissue were dyed with 1.5% 2,3.5-triphenyl tetrazolium chloride (TTC) for 15 min, and monitored under a digital camera. The images obtained were analyzed with Image J, and the infarct area was calculated (mm^2^). The infarct volume (mm^3^) was estimated according to the following equation (Equation (2))

(2) Infarct volume (mm^3^) = Area_A_ × 2 + Area_B_ × 2 + Area_C_ × 2

### 4.6. Neurological Deficits

Mice were tested for neurological deficits 72 h after middle cerebral arterial occlusion followed by reperfusion (MCAO/reperfusion). Scoring was based on the method of Hara *et al.* [39]. The scale is as follows: 0, no observable neurological deficits (normal); 1, closing of the left eye; 2, failure to extend the right foreleg; 3, walking to the contralateral side; 4, loss of righting reflex or moving. The each mouse belonged was masked for the investigator.

### 4.7. Assay of Plasma CLZ Concentrations

On the day before the administration of CLZ solution or dispersions, the rats was anesthetized by isoflurane (2.5%), and a silicone tubing (I.D. 0.5 mm, O.D. 1.0 mm) was cannulated into the right jugular vein. The next day, 0.3 mL of CLZ solution or dispersions (0.1 mg/kg) was injected into the femoral vein, and venous blood (200 μL) was collected through the cannula at 0, 5, 10, 15, 20, 30, 45, 60, 90 and 120 min after the single injection of CLZ. The blood was centrifuged at 3000 rpm for 20 min at 4 °C, and the plasma obtained was stored at −80 °C until CLZ analysis by the HPLC method described above. The CLZ concentration was analyzed according to Equation (3):

(3) $$C_{\text{CLZ}}=A\cdot e^{-\alpha \cdot t}+B\cdot e^{-\beta \cdot t}$$

where *t* is time (0–120 min) after CLZ injection, and *C*_CLZ_ is the concentration of CLZ at the corresponding time. α and β show the elimination rate constants in the first and second-phases, and A and B are the CLZ concentrations in the α- and β-phases, respectively. A nonlinear least-squares computer program (MULTI) was employed for the calculation.

The area under the CLZ concentration-time curve (*AUC*_CLZ_), area under the first moment curve (*AUMC*_CLZ_) and mean residence time (*MRT*_CLZ_) were estimated by using Equations (4)–(6).

(4) $${AUC}_{\text{CLZ}}=\int_{0\text{min}}^{120\text{min}}C_{\text{CLZ}}dt$$

(5) $${AUMC}_{\text{CLZ}}=\int_{0\text{min}}^{120\text{min}}C_{\text{CLZ}}\cdot tdt$$

(6) $${MRT}_{\text{CLZ}}=\frac{{AUMC}_{\text{CLZ}}}{{AUC}_{\text{CLZ}}}$$

Briefly, *AUC*_CLZ_ was determined according to the trapezoidal rule.

### 4.8. CLZ Concentration in Brain

Focal cerebral ischemia/reperfusion was induced by MCAO on the left side as described above. Three days after reperfusion, the brain was removed, and slices 1 mm anterior and 1 mm posterior to the center of the bregma were cut using Brain Matrices (Brain Science Idea Co., Ltd., Osaka, Japan). The sliced brain tissue was divided at the center, and the right and left parts were homogenized separately in methanol. The homogenates were centrifuged at 10,000 rpm for 15 min at 4 °C, and the resultant supernatants were used for the measurement of CLZ concentration. The CLZ concentration was analyzed by the HPLC method described above.

### 4.9. Measurement of Blood Pressure

Blood pressure was measured according to previously published methods [40]. Briefly, rats were anesthetized with Urethane (1.2 g/kg, i.p.) and restrained on a board in a supine position. Blood pressure was measured for 140 min (11:00–13:20). A polyethylene cannula tube (PE50, Clay Adams, Division of Becton Dickinson and Company, Parsippany, NJ, USA) was cannulated into the left side of common carotid artery, and a pressure transducer (DX312, Nihon Kohden, Shinjuku, Tokyo) was connected in the other end of the polyethylene cannula tubeto measure the blood pressure. To prevent blood coagulation in the cannula, 7.7 μg/mL heparin solution was injected at a speed of 0.38 mL/h. The board was kept warm at about 37 °C to maintain the rat body temperature.

### 4.10. Measurement of Blood Flow in Carotid Artery and Brain

Rats were anesthetized with Urethane (1.2 g/kg, i.p.), and blood flow in carotid artery and brain was measured by non-contact type laser doppler flowmetry (OMEGA Flow FLO-N1, OMEGAWAVE INC., Tokyo, Japan). For the measurement of blood flow in the carotid artery, a non-contact type probe (GJ probe) connected to the laser doppler flowmetry (FLO-N1) was set on the left carotid artery, and measurements were collected for 140 min. For the measurement of blood flow in brain tissue, a hole 1 mm in diameter was made using an engraving drill into the area 1.0 mm caudal and 3.5 mm to the left aspect from the bregma. A connect probe non-contact type connect probe (GJ probe) was passed through the hole (depth, 1.4 mm), and blood flow in the brain tissue was measured for 30 min. The board was kept warm at about 37 °C to maintain the rat body temperature.

### 4.11. Statistical Analysis

All values are presented as mean ± standard deviation (S.D.) or standard error of the mean (S.E.). Unpaired Student’s *t*-test was used to evaluate statistical differences, and multiple groups were evaluated by one-way analysis of variance followed by Dunnett’s multiple comparison. *p* values less than 0.05 were considered significant.

## 5. Conclusions

In the present study, we attempted to prepare novel drug nanoparticles for use in preparing a CLZ formulation for intravenous administration without the use of a toxic solvent, such as dimethyl sulfoxide, and succeeded in preparing a high quality dispersion containing CLZ nanoparticles (particle size, 81 ± 59 nm, means ± S.D.). The dispersion containing CLZ nanoparticles ameliorates neurological deficits due to ischemic stroke in MCAO/reperfusion mice. In addition, at concentrations of 0.1–0.6 mg/kg, the intravenous administration of the dispersions containing CLZ nanoparticles does not affect blood pressure or flow under normal conditions, and the plasma pharmacokinetics is similar to that of the CLZ solution. It is possible that dispersions containing CLZ nanoparticles will provide effective therapy for ischemic stroke patients, and that injection preparations of lipophilic drugs using drug nanoparticles expand their usage for therapy. This study provides important information to develop further studies aimed at developing therapies for ischemic stroke patients, and to develop less toxic injection preparations.

## References

[1] Go A.S. (2005). The epidemiology of atrial fibrillation in elderly persons: The tip of the iceberg. Am. J. Geriatr. Cardiol..

[2] Nito C., Kamada H., Endo H., Niizuma K., Myer D.J., Chan P.H. (2008). Role of the p38 mitogen-activated protein kinase/cytosolic phospholipase A2 signaling pathway in blood-brain barrier disruption after focal cerebral ischemia and reperfusion. J. Cereb. Blood Flow Metab..

[3] Diaz-Ruiz A., Vacio-Adame P., Monroy-Noyola A., Méndez-Armenta M., Ortiz-Plata A., Montes S., Rios C. (2014). Metallothionein-II inhibits lipid peroxidation and improves functional recovery after transient brain ischemia and reperfusion in rats. Oxid. Med. Cell. Longev..

[4] Castillo J., Dávalos A., Noya M. (1997). Progression of ischaemic stroke and excitotoxic aminoacids. Lancet.

[5] Cuzzocrea S., Riley D.P., Caputi A.P., Salvemini D. (2001). Antioxidant therapy: A new pharmacological approach in shock, inflammation, and ischemia/reperfusion injury. Pharmacol. Rev..

[6] The National Institute of Neurological Disorders and Stroke rt-PA Stroke Study Group (1995). Tissue plasminogen activator for acute ischemic stroke. N. Engl. J. Med..

[7] Ishiguro M., Mishiro K., Fujiwara Y., Chen H., Izuta H., Tsuruma K., Shimazawa M., Yoshimura S., Satoh M., Iwama T. (2010). Phosphodiesterase-III inhibitor prevents hemorrhagic transformation induced by focal cerebral ischemia in mice treated with tPA. PLoS ONE.

[8] Liu Y., Shakur Y., Yoshitake M., Kambayashi J.-I. (2001). Cilostazol (pletal®): A dual inhibitor of cyclic nucleotide phosphodiesterase type 3 and adenosine uptake. Cardiovasc. Drug Rev..

[9] Kim K.Y., Shin H.K., Choi J.M., Hong K.W. (2002). Inhibition of lipopolysaccharide induced apoptosis by cilostazol in human umbilical vein endothelial cells. J. Pharmacol. Exp. Ther..

[10] Fujita Y., Lin J.X., Takahashi R., Tomimoto H. (2008). Cilostazol alleviates cerebral small-vessel pathology and white-matter lesions in stroke-prone spontaneously hypertensive rats. Brain Res..

[11] Tanaka K., Gotoh F., Fukuuchi Y., Amano T., Uematsu D., Kawamura J., Yamawaki T., Itoh N., Obara K., Muramatsu K. (1989). Effects of a selective inhibitor of cyclic AMP phosphodiesterase on the pial microcirculation in feline cerebral ischemia. Stroke.

[12] Oyama N., Yagita Y., Kawamura M., Sugiyama Y., Terasaki Y., Omura-Matsuoka E., Sasaki T., Kitagawa K. (2011). Cilostazol, not aspirin, reduces ischemic brain injury via endothelial protection in spontaneously hypertensive rats. Stroke.

[13] Kasahara Y., Nakagomi T., Matsuyama T., Stern D., Taguchi A. (2012). Cilostazol reduces the risk of hemorrhagic infarction after administration of tissue-type plasminogen activator in a murine stroke model. Stroke.

[14] Nonaka Y., Tsuruma K., Shimazawa M., Yoshimura S., Iwama T., Hara H. (2009). Cilostazol protects against hemorrhagic transformation in mice transient focal cerebral ischemia-induced brain damage. Neurosci. Lett..

[15] Ishida T., Kiwada H. (2004). Accelerated blood clearance of PEGylated liposomes after repeated injection. Drug Deliv. Syst..

[16] Kataoka K., Harada A., Nagasaki Y. (2001). Block copolymer micelles for drug delivery design, characterization and biological significance. Adv. Drug Deliv. Rev..

[17] Igarashi R., Takenaga M., Takeuchi J., Kitagawa A., Matsumoto K., Mizushima Y. (2001). Marked hypotensive and blood flow-increasing effects of a newlipo-PGE1 (lipo-AS013) due to vascular wall targeting. J. Control. Release.

[18] Murakami H., Kobayashi M., Takeuchi H., Kawashima Y. (2000). Further application of a modified spontaneous emulsification solvent diffusion method to various types of PLGA and PLA polymers for preparation of nanoparticles. Powder Technol..

[19] Kawashima Y. (2006). Design of poly(lactic-*co*-glycolic acid) (PLGA) nanosphere for developing to DDS. J. Pharm. Sci. Technol. Jpn..

[20] Tsukada Y., Hara K., Bando Y., Huang C.C., Kousaka Y., Kawashima Y., Morishita R., Tsujimoto H. (2009). Particle size control of poly(d,l-lactide-*co*-glycolide) nanospheres for sterile applications. Int. J. Pharm..

[21] Nagai N., Ito Y., Okamoto N., Shimomura Y. (2014). A nanoparticle formulation reduces the corneal toxicity of indomethacin eye drops and enhances its corneal permeability. Toxicology.

[22] Nagai N., Yoshioka C., Mano Y., Tanabe W., Ito Y., Okamoto N., Shimomura Y. (2015). A nanoparticle formulation of disulfiram prolongs corneal residence time of the drug and reduces intraocular pressure. Exp. Eye Res..

[23] Carmichael S.T. (2005). Rodent models of focal stroke: size, mechanism, and purpose. NeuroRx.

[24] Wells M.R., Kraus K., Batter D.K., Blunt D.G., Weremowitz J., Lynch S.E., Antoniades H.N., Hansson H.A. (1997). Gel matrix vehicles for growth factor application in nerve gap injuries repaired with tubes: A comparison of biomatrix, collagen, and methylcellulose. Exp. Neurol..

[25] Gupta D., Tator C.H., Shoichet M.S. (2006). Fast-gelling injectable blend of hyaluronan and methylcellulose for intrathecal, localized delivery to the injured spinal cord. Biomaterials.

[26] Chouly C., Pouliquen D., Lucet I., Jeune J.J., Jallet P. (1996). Development of superparamagnetic nanoparticles for MRI: Effect of particle size, charge and surface nature on biodistribution. J. Microencapsul..

[27] Begley D.J. (2004). Delivery of therapeutic agents to the central nervous system: The problems and the possibilities. Pharmacol. Ther..

[28] Dobrovolskaia M.A., Aggarwal P., Hall J.B., McNeil S.E. (2008). Preclinical studies to understand nanoparticle interaction with the immune system and its potential effects on nanoparticle biodistribution. Mol. Pharm..

[29] Toy R., Peiris P.M., Ghaghada K.B., Karathanasis E. (2014). Shaping cancer nanomedicine: The effect of particle shape on the *in vivo* journey of nanoparticles. Nanomedicine.

[30] Kimura Y., Tani T., Kanbe T., Watanabe K. (1985). Effect of cilostazol on platelet aggregation and experimental thrombosis. Arzneim. Forsch..

[31] Minami N., Suzuki Y., Yamamoto M., Kihira H., Imai E., Wada H., Kimura Y., Ikeda Y., Shiku H., Nishikawa M. (1997). Inhibition of shear stress-induced platelet aggregation by cilostazol, a specific inhibitor of cGMP-inhibited phosphodiesterase, *in vitro* and *ex vivo*. Life Sci..

[32] Kohda N., Tani T., Nakayama S., Adachi T., Marukawa K., Ito R., Ishida K., Matsumoto Y., Kimura Y. (1999). Effect of cilostazol, a phosphodiesterase III inhibitor, on experimental thrombosis in the porcine carotid artery. Thromb. Res..

[33] Kawamura K., Watanabe K., Kimura Y. (1985). Effect of cilostazol, a new antithrombotic drug, on cerebral circulation. Arzneim. Forsch..

[34] Lee J.H., Park S.Y., Lee W.S., Hong K.W. (2005). Lack of antiapoptotic effects of antiplatelet drug, aspirin and clopidogrel, and antioxidant, MCI-186, against focal ischemic brain damage in rats. Neurol. Res..

[35] Lee J.H., Kim K.Y., Lee Y.K., Park S.Y., Kim C.D., Lee W.S., Rhim B.Y., Hong K.W. (2004). Cilostazol prevents focal cerebral ischemic injury by enhancing casein kinase 2 phosphorylation and suppression of phosphatase and tensin homolog deleted from chromosome 10 phosphorylation in rats. J. Pharmacol. Exp. Ther..

[36] Lee J.H., Park S.Y., Shin H.K., Kim C.D., Lee W.S., Hong K.W. (2008). Protective effects of cilostazol against transient focal cerebral ischemia and chronic cerebral hypoperfusion injury. CNS Neurosci. Ther..

[37] Wakida K., Morimoto N., Shimazawa M., Hozumi I., Nagase H., Inuzuka T., Hara H. (2006). Cilostazol reduces ischemic brain damage partly by inducing metallothionein-1 and -2. Brain Res..

[38] Okamoto N., Ito Y., Nagai N., Murao T., Takiguchi Y., Kurimoto T., Mimura O. (2010). Preparation of ophthalmic formulations containing cilostazol as an anti-glaucoma agent and improvement in its permeability through the rabbit cornea. J. Oleo Sci..

[39] Hara H., Huang P.L., Panahian N., Fishman M.C., Moskowitz M.A. (1996). Reduced brain edema and infarction volume in mice lacking the neuronal isoform of nitric oxide synthase after transient MCA occlusion. J. Cereb. Blood Flow Metab..

[40] Funakami Y., Hata T., Itoh E., Itano S. (2007). Effects of some β-adrenoceptor antagonists on orthostatic hypotension in repeatedly cold- (SART-) stressed rats. Biol. Pharm. Bull..

